# Factors associated with pharmacological and psychotherapy treatments adherence in patients with borderline personality disorder

**DOI:** 10.3389/fpsyt.2022.1056050

**Published:** 2022-12-13

**Authors:** Motahareh Sadat Mirhaj Mohammadabadi, Homa Mohammadsadeghi, Mehrdad Eftekhar Adrebili, Zahra Partovi Kolour, Fatemeh Kashaninasab, Vahid Rashedi, Mohammadreza Shalbafan

**Affiliations:** ^1^Mental Health Research Center, Psychosocial Health Research Institute, Department of Psychiatry, School of Medicine, Iran University of Medical Sciences, Tehran, Iran; ^2^Students Research Committee, School of Medicine, Iran University of Medical Sciences, Tehran, Iran; ^3^Iranian Research Center on Aging, Department of Aging, University of Social Welfare and Rehabilitation Sciences, Tehran, Iran

**Keywords:** borderline personality disorder, treatment outcome, pharmacotherapy, psychotherapy, personality disorders

## Abstract

**Background:**

Borderline personality disorder is a major mental illness characterized by sustained relationship instability, impulsive behavior, and intense affects. Adherence is a complex behavior, from minor refusals to abandonment of treatment, which can be affected by various factors. Therefore, the present study aimed to investigate the factors affecting pharmacological and psychotherapy adherence, patients' attitude toward medication, and assessing medication and treatment adherence in patients with borderline personality disorder referred to an outpatient referral clinic in Tehran, Iran.

**Methods:**

The study was a cross-sectional study. The files of patients with borderline personality disorder referred to the outpatient clinic of the Tehran Psychiatric Institute were reviewed as the first step. Data were collected using the Drug Attitude Inventory-10 (DAI-10) questionnaire and a questionnaire to determine the attitude of patients toward pharmacological and psychotherapy treatment as well as therapeutic adherence. After collecting data, patients' therapeutic adherence was divided into poor, partial, and good compliance.

**Results:**

Ninety-four patients were involved in the study, and fifty-four were women. Findings of DAI showed that 54 (57.4%) participants had negative attitudes toward medication, while 38 (40.4%) participants showed a negative attitude toward psychotherapy treatment. Additionally, the percentage of patients with good psychotherapy adherence (44.7%) was higher than that of patients with good medication adherence (31.9%). The most common reasons for discontinuation of treatment were medication side effects (53.1%), dissatisfaction with the therapist (40.3%), and then fear of medication dependence (40%). Patients with higher education levels and a positive history of hospitalization in a psychiatric ward had better adherence to psychotherapy (*P* < 0.05).

**Conclusion:**

Results of the current study show that attitude toward psychotherapy is more favorable than pharmacotherapy among patients with BPD. The rationale may be that medications are mainly prescribed for comorbid conditions and do not have substantial effects on the BPD symptoms, resulting in low medication adherence.

## Background

Borderline personality disorder (BPD) is characterized by severe instability in impulsive feelings, identities, relationships, and behaviors (1). BPD is a major mental illness with a prevalence of ~1–3% among the general population. The instability of relationships, the creation, and regulation of impulses, self-awareness, and a sense of agency, were some of the main features of the disorder (2). BPD is a severe disorder that accounts for 20 to 40% of psychiatric admissions, and it is estimated that 84% of patients show suicidal behaviors, and 8% of them die due to suicide (3, 4). The exact etiology of BPD is still unclear and is likely multifactorial and heterogeneous; current explanations assume the stress-diathesis model, with an interaction between the experience of traumatic events during childhood (e.g., sexual abuse, neglect) and genetic factors (5). BPD causes many problems for the patient and the community. The repeated and severe damages caused by this disorder affect an individual's entire life, including occupational problems, dropout, disruption of relationships with friends, colleagues, and family members, failure in marriage, and high-risk and unstable sexual relations. Almost 11–69% of patients are substance users, 22% suffer from alcoholism, and 25–50% of female prisoners are patients with BPD (6–11).

In a study by Bellino et al. patients with more severe BPD and higher symptoms such as fear of abandonment, emotional instability, and identity showed a better chance of combining psychotherapy with fluoxetine (12). A systematic review and meta-analysis by Gartlehner et al. concluded that despite the common use of pharmacotherapies for patients with BPD, the available evidence does not support the efficacy of pharmacotherapies alone in reducing the severity of BPD (13). Although psychotherapy is still the first line of treatment for patients with BPD, medications are commonly prescribed for them. The use of antidepressants has decreased, and mood stabilizers and second-generation antipsychotics have increased (14).

Adherence is a complex behavior, from minor refusals of treatment to abandonment of therapy, which can be affected by various factors (15). Five areas were observed that were configured as factors influencing adherence: personal, systemic, disease-related, sociodemographic, and treatment-related factors (16). Rejection of treatment is often associated with irrational fears and misjudgments; the most common cause is fear of medication dependence. Various studies have been performed on patients with personality disorders, schizophrenia, and bipolar, showing that one of the critical issues in the treatment is adherence to treatment by patients (17, 18). Due to the prevalence of BPD and the need for long-term pharmacological and psychotherapy treatment, the high cost of treatment imposed on the health care system, and the fact that no study was conducted in Iran, it seems necessary to evaluate the medication and psychotherapy adherence in these patients. Hoping that by working on changeable factors, patients' adherence and, consequently, their function will improve, and the recurrence of symptoms and re-admission will be reduced.

An accurate prevalence rate of BPD is not available in Iran. Due to the cultural and civil context in Iran, and the existence of religious thoughts in the Iranian community and families, in most cases, some behaviors of patients with BPD, such as extramarital relationships and substance use, are contrary to social norms and severely complicate among Iranian society (19, 20). In the present study, we investigate the factors of rejection and reasons for discontinuation of treatment, more specifically psychotherapy as the first-line treatment of BPD, to provide a way to improve adherence in these patients and to introduce practical methods into the clinical practice of therapists in future studies.

## Methods

### Study design and participants

The study was conducted as a cross-sectional study in 2020. In this study, the files of patients with BPD who were referred to the outpatient adult psychiatric clinic of Tehran Psychiatric Institute, Tehran, Iran, from April 2014 to April 2019 were reviewed as the first step.

Patient information including name, gender, age, occupation, education, marital status, family history of psychiatric illness, type of treatment, history of substance use, history of hospitalization, history of suicide and self-harm, comorbidity, and medication side effects on the files, was recorded. In the next step, patients were contacted *via* phone to collect the information, including receiving or not receiving medical or psychotherapy treatment, last referral date, confirmation of demographic data including any change of them through time, current life status (alone, with family), suicide and self-harm, financial status, legal issues, medication side effects, and location (far or close to the treatment center). In addition, they were asked to fill out the Drug Attitude Inventory (DAI) questionnaire and a researcher-made questionnaire to determine the attitude of patients toward pharmacological and psychotherapy treatment and therapeutic adherence.

Patients who did not respond were called three times within a week, and if they did not respond, they were given a message and excluded from the study if they did not respond again.

### Instruments

In addition to demographic items, two questionnaires were used in this study. The first questionnaire was DAI-10 based, which evaluates the attitude of patients toward medication with 10 yes-no questions, and a higher total score between +10 and −10 shows a more positive attitude. Shariati et al. confirmed the psychometric properties of the Persian version of the scale. They reported that Test-retest reliability was 0.805 and Cronbach's α 0.787. The concurrent validity between the DAI-10 score and Medication possession rate (MPR) at the study time was 0.676, and the positive predictive value of the DAI-10 score for medication compliance at assessment time was about 88.9%. The Spearman correlation coefficient between the two tests was 0.822 [95% confidence interval (CI): (0.652–0.901)]. They also reported a cut-off point +1, with a specificity of 81.5% and a sensitivity of 89.1% (21).

Additionally, based on the participants‘ reports, the researcher evaluated the patients' current medical adherence, and according to the MPR, patients were divided into three groups: poor, good, and partial pharmacological adherence. Based on MPR, more than 80% equal good adherence, 50–80% equal partial adherence, and less than 50% equal poor adherence. In our study last month, the MPR represents the proportion of days of medication supply in a specified period and is given by the sum of days divided by the number of days within the time interval (22, 23). In addition, based on the participant‘s report and documented visits for psychotherapy sessions, the psychotherapy adherence of the patients was evaluated. The division into poor, partial, and good psychotherapy adherence is based on the percentage of on-time performed sessions to proposed sessions by the therapist in the last month, and the cut-offs were similar to MPR.

To the best of our knowledge, there is no comprehensive, validated Persian questionnaire to evaluate the attitude of patients toward medication and psychotherapy treatment and reasons for poor/partial drug compliance. Therefore, the second questionnaire was a researcher-made questionnaire to determine the attitude of patients toward pharmacological and psychotherapy treatment and therapeutic adherence. This questionnaire consisted of 17 questions developed by the authors and validated by eight psychiatric faculty members of the Iran University of Medical Sciences. To standardize and validate the questionnaire, a qualitative assessment was done using an expert panel, and a quantitative assessment was done using the content validity ratio (CVR) and content validity index (CVI). According to the overall CVR (0.75 <) and the CVI (0.79 <), the questionnaire had content validity (24). The reliability of this questionnaire was checked in this study and Cronbach's α was 0.82. This questionnaire was divided into two parts to emphasize reasons for poor/partial medication compliance. The first part, which included questions (1–6), was filled by all 94 patients, and if the participants‘ medication adherence were poor/ partial, based on MPR, they were asked to fill the second part (questions 7–17). Therefore, 64 patients filled out the second part of the questionnaire. All questionnaires were filled out, and evaluations were performed for each participant on the same day.

### Statistical analysis

Data were analyzed by SPSS 22 (IBM Inc, New York, USA). Data are presented as numbers and percentages and compared between groups by chi-square and *t*-test. *P*-values < 0.05 were assumed as statistically significant. Type I error (α) is set at 5%.

## Results

Ninety-four patients were involved in the study; the mean age was 33.6, 54 (57.4%) were female, and the others were male. Demographic data, medication and psychotherapy adherence, and psychiatric history of the participants are shown in Table 1.

**Table 1:** Demographic and clinical characteristics of participants.

**Variable**		**%**	***n***
Employment	Employed	41	43.6
	College students	10	10.6
	Unemployed	43	45.7
Education	Under diploma	21	22.3
	Diploma and higher	43	45.8
	Bachelor's degree	21	22.3
	Master's degree or higher	9	9.6
Marital status	Single	49	52.1
	Married	32	34
	Divorced	12	12.8
Medication Adherence	Poor	46	48.9
	Moderate	18	19.1
	Good	30	31.9
Psychotherapy Adherence	Poor	21	22.3
	Partial	31	33
	Good	42	44.7
Psychiatric Hospitalization		21	22.3
Family history of psychiatric illness		34	36.2
Legal problems		15	16
Self-injury		30	31.9
Suicide attempt		40	42.6
Illegal substance use		42	44.7

Answers to the researcher-made questionnaire about the attitude of patients toward pharmacological and psychotherapy treatment and therapeutic adherence are shown in Table 2, and reasons for poor/partial drug compliance are shown in Table 3.

**Table 2:** Insight and attitude toward treatment (First part of the questionnaire).

**Number**	**Questions**	**Answer**	**n (%)**
**1**	I am sick and need treatment	Yes	65 (69.1)
		No	29 (30.9)
**2**	Lack of treatment can disrupt my life	Yes	59 (62.8)
	and the lives of those around me	No	35(37.2)
**3**	Lack of treatment can cause problems	Yes	67(71.3)
	in my relationships with others	No	27(28.7)
**4**	My illness is treatable	Yes	73(77.7)
		No	21(22.3)
**5**	Medication has not helped my condition	Yes	49(52.1)
		No	45(47.9)
**6**	Psychotherapy or counseling did not	Yes	38(40.4)
	help my condition	No	56(59.6)

**Table 3:** Reasons for poor/partial drug compliance (Second part of the questionnaire).

**Questions**	**Number**	**Answer**	***n*** **(%)**
I did not continue the treatment due to	**1**	Yes	12 (18.8)
the cost of the doctor's visit.		No	52 (81.2)
I did not go to another center due to	**2**	Yes	10 (15.6)
travel expenses.		No	54 (84.4)
Because I was not satisfied with my	**3**	Yes	26 (40.6)
doctor, I did not see him again.		No	38 (59.4)
Because I could not easily make an	**4**	Yes	18 (28.1)
appointment, I did not return.		No	46 (71.9)
The misbehavior of others and their talk	**5**	Yes	12 (18.8)
about my illness caused me to stop taking my medication.		No	52 (81.3)
Medication side effects caused me to	**6**	Yes	34 (53.1)
stop my medication.		No	30 (46.9)
I stopped taking the medication because	**7**	Yes	13 (20.3)
of the cost of the medication.		No	51 (79.7)
Because the duration of my medication	**8**	Yes	21 (32.8)
was long, I did not take any more medications.		No	43 (67.2)
I stopped taking it for fear of medication	**9**	Yes	26 (40.6)
dependence.		No	38 (59.4)
I stopped psychotherapy due to the cost	**10**	Yes	21 (32.8)
of psychotherapy.		No	43 (67.2)
Long-term psychotherapy was the	**11**	Yes	13 (20.3)
reason for interrupting psychotherapy sessions.		No	51 (79.4)

Moreover, regarding the DAI-10 total score, 54 patients (57.4%) had a negative attitude, and 40 patients (42.6%) had a positive attitude toward the medication.

There was no relationship among gender (*P* = 0.33), job (*P* = 0.16), education (*P* = 0.71), marital status (*P* = 0.37), history of psychiatric hospitalization (*P* = 0.81), history of self-injury (*P* = 0.83), suicide history (*P* = 0.54), history of substance use (*P* = 0.85), comorbidity with psychiatric disorders (*P* = 0.63), family history of mental illness (*P* = 0.77) and medication adherence.

There was no relationship among gender (*P* = 0.62), job (*P* = 0.30), marital status (*P* = 0.75), history of self-injury (*P* = 0.36), suicide history (*P* = 0.64), history of substance use (*P* = 0.57), comorbidity with psychiatric disorders (*P* = 0.23), family history of mental illness (*P* = 0.52) and type of medication used (*P* = 0.06) and psychotherapy adherence.

There was a statistically significant relationship between education level and psychotherapy adherence (*P* = 0.02). The best adherence to psychotherapy was obtained in postgraduate education (with 66% good adherence among this subgroup), and the worst medication adherence in undergraduate education (with 22% good adherence among this subgroup; *P* = 0.04). The history of psychiatric hospitalization has a significant relationship with psychotherapy adherence (*P* = 0.01); those who had a history of hospitalization had better adherence to psychotherapy than non-hospitalized patients (66 vs. 36.9% of the relevant subgroup).

Based on the results, patients' belief that their disorder is treatable, lack of treatment disrupts their lives, and those around them are inversely related to poor and relative medication adherence. Patients with a negative medication attitude are 1.3 times more likely to have poor medication adherence and 2.8 times more likely to have relative medication adherence. Our finding revealed that patients with postgraduate education are 2.2 times more likely to have good psychotherapy adherence.

There was a significant relationship between attitudes toward medication and the belief that “lack of treatment disrupts relationships with others”. 87.5% of patients with a positive attitude stated that lack of treatment could disrupt their relationships with others. 81.5% of patients who disagreed with this view had a negative medication attitude (*P* = 0.03). There was a significant relationship between attitudes toward medication and the belief that “their disorder can be treated”. 95% of patients with a positive medication attitude believed their disorder could be treated (*P* = 0.001). 72.2% of patients with a negative attitude toward medication believed that its side effects had interrupted their treatment (*P* = 0.02).

## Discussion

This study aimed to determine the factors contributing to pharmacological and psychotherapy adherence in 94 patients with BPD who were referred to the outpatient clinic of Tehran Psychiatric Institute. The lack of adherence to treatment is one of the most important mental health problems and has come to be called the “invisible epidemic”.

This study showed that gender, occupation, education, marriage, history of psychiatric hospitalization, history of self-harm and suicide attempt, and history of substance use had no significant relationship with medication adherence. However, education and history of hospitalization were significantly higher in patients with good adherence to psychotherapy. The finding is consistent with findings of past studies by Caqueo-Urízar et al. (25) and Winton-Brown et al. (26) which indicated that a lower level of adherence to pharmacological treatment has been observed in patients belonging to an ethnic minority or with a lower educational level (25, 26).

In 2020, Zarei et al. (27) examined BPD improvement at different time intervals of transference-focused treatment. Their findings showed that gender, occupation, education, marriage, history of psychiatric hospitalization, and history of self-harm had no significant relationship with adherence to treatment, which was consistent with our results (27). In another study, there was no significant relationship between the history of self-harm, substance use, psychiatric comorbidity, history of psychiatric illness, type of medication, and medication adherence that was consistent with our results (28).

In a study among soldiers, Soltaninejad et al. showed that in patients with BPD, self-injury, substance use, psychiatric comorbidity, history of psychiatric illness, and type of pharmacotherapy had no significant relationship with adherence to medication that was consistent with our results (29).

The findings of our study showed better attitudes toward psychotherapy than medication. On the other hand, negative attitudes toward medication and fear of medication dependence were the most common reason for poor medication adherence. In addition, 42.6% of participants had positive attitudes toward medications in the current study, and 31.9% had good medication adherence. It‘s lower than the percentage with the same scales, DAI-10 and MPR, among some other psychiatric patients. Asadi et al. reported that more than 85% of participants had positive attitudes toward medication and more than 55% had good medication adherence among Iranian male patients with methamphetamine-induced psychotic disorder in a three-month follow-up study after discharge from a mental hospital (22).

Similarly, Azadforouz et al. (30) reported that 63.8% of patients with bipolar I disorder had good medication adherence in a 6-month follow-up study in Iran (30). A plausible rationale for lower medication adherence and less positive attitude toward medication among patients with BPD is the lack of substantial effectiveness of medications in this disorder, and probably lack of positive previous experience with medication which is not proven for BPD, despite pharmacotherapy is still highly prevalent in the management of the patients (31). These findings emphasize the importance of psychotherapeutic approaches for managing patients with BPD as a more effective and accepted treatment method than pharmacotherapy.

## Limitations

There were several limitations; the first one was the small sample size. Also, some inaccurate recording of documents, the lack of facilities to follow up with patients, and failure to assess the severity of the disorder as well sub-threshold comorbidity traits, discrimination between different psychotherapeutic approaches, and emphasizing on change of therapist as a cause of non-adherence were other limitations. In addition, the lack of a control group is another limitation of the current study. The nature of the study and retrospective gathering of some of the data could be pointed to as other study limitations. Moreover, our findings cannot show a causality effect between psychosocial variables and primary outcomes.

It is suggested to consider these limitations for future studies and perform a multi-centered study with the control group, follow-up, and evaluation of the severity of symptoms. Further evaluation of the psychometric properties of the researcher-made questionnaire is also recommended. Future studies on interventions to improve psychotherapy adherence are suggested too.

## Conclusion

In conclusion, the current study's findings showed that most patients with BPD had negative attitudes toward medication and medication adherence among them was lower than psychotherapy adherence. The rationale may be that medications are mainly prescribed for other comorbid conditions and do not substantially affect the BPD symptoms, resulting in low medication adherence. We also found that the education level of the patients had a positive relationship with good psychotherapy adherence. Therefore, patients with lower education levels should be followed-up by healthcare providers to prevent discontinuation of their psychotherapy sessions.

Overall, the findings emphasize the importance of psychotherapy, as the first-line treatment approach, among patients with BPD and suggest future studies on interventions to improve psychotherapy adherence of the patients.

## Data availability statement

The raw data supporting the conclusions of this article will be made available by the authors, without undue reservation.

## Ethics statement

The studies involving human participants were reviewed and approved by the Institutional Review Board of Iran University of Medical Sciences. The patients/participants provided their written informed consent to participate in this study.

## Author contributions

MM, HM, ME, and MS: conceptualization and design. MM, ZP, and FK: data gathering. MM, HM, VR, and MS: initial draft preparation. MM, HM, ME, ZP, FK, VR, and MS: editing and review. All authors read and approved the final manuscript.

## References

[1] Falconer CJ Cutting P Davies EB Hollis C Stallard P Moran P. Adjunctive avatar therapy for mentalization-based treatment of borderline personality disorder: a mixed-methods feasibility study. Evid Based Ment Health. (2017) 20:123–7. 10.1136/eb-2017-102761 29056609 PMC5750410

[2] Colle L Hilviu D Rossi R Garbarini F Fossataro C. Self-harming and sense of agency in patients with borderline personality disorder. Front Psychiatry. (2020) 11:449. 10.3389/fpsyt.2020.00449 32547429 PMC7273851

[3] Sadock BJ. Kaplan & Sadock's Synopsis of Psychiatry: Behavioral Sciences/Clinical Psychiatry. Philadelphia, PA: Lippincott Williams & Wilkins (2016).

[4] Pompili M Girardi P Ruberto A Tatarelli R. Suicide in borderline personality disorder: a meta-analysis. Nord J Psychiatry. (2005) 59:319–24. 10.1080/08039480500320025 16757458

[5] Belsky DW Caspi A Arseneault L Bleidorn W Fonagy P Goodman M. Etiological features of borderline personality related characteristics in a birth cohort of 12-year-old children. Dev Psychopathol. (2012) 24:251–65. 10.1017/S0954579411000812 22293008 PMC3547630

[6] O'Donohue W Fowler KA Lilienfeld SO. Personality Disorders: Toward the DSM-V. New York, NY: Sage Publications (2007).

[7] American-Psychiatric-Association. Diagnostic and Statistical Manual of Mental Disorders. Washington, DC: American Psychiatric Publishing (2013).

[8] Gunderson JG. Borderline Personality Disorder: A Clinical Guide. Washington, DC: American Psychiatric Publishing (2009).

[9] Sansone RA Sansone LA. Borderline personality and criminality. Psychiatry. (2009) 6:16–20. PMC2790397 20011575

[10] Keuroghlian AS Zanarini MC. Lessons Learned From Longitudinal Studies of Personality Disorder. Washington, DC: American Psychiatric Publishing (2015).

[11] Zanarini MC Frankenburg FR Reich DB Silk KR Hudson JI McSweeney LB. The subsyndromal phenomenology of borderline personality disorder: a 10-year follow-up study. Am J Psychiatry. (2007) 164:929–35. 10.1176/ajp.2007.164.6.929 17541053

[12] Bellino S Bozzatello P Bogetto F. Combined treatment of borderline personality disorder with interpersonal psychotherapy and pharmacotherapy: predictors of response. Psychiatry Res. (2015) 226:284–8. 10.1016/j.psychres.2014.12.064 25677397

[13] Gartlehner G Crotty K Kennedy S Edlund MJ Ali R Siddiqui M. Pharmacological treatments for borderline personality disorder: a systematic review and meta-analysis. CNS Drugs. (2021) 35:1053–67. 10.1007/s40263-021-00855-4 34495494 PMC8478737

[14] Starcevic V Janca A. Pharmacotherapy of borderline personality disorder: replacing confusion with prudent pragmatism. Curr Opin Psychiatry. (2018) 31:69–73. 10.1097/YCO.0000000000000373 29028643

[15] Tanesi PHV Yazigi L. Fiore MLdM, Pitta JCdN. Compliance in the treatment of borderline personality disorders. Estudos de Psicologia. (2007) 12:71–8. 10.1590/S1413-294X2007000100009

[16] Caqueo-Urízar A Urzúa A Mena-Chamorro P Bravo De la Fuente J. Effects of adherence to pharmacological treatment on the recovery of patients with Schizophrenia. Healthcare. (2021) 9:1230. 10.3390/healthcare9091230 34575005 PMC8468521

[17] Greene M Paladini L Lemmer T Piedade A Touya M Clark O. Systematic literature review on patterns of pharmacological treatment and adherence among patients with bipolar disorder type I in the USA. Neuropsychiatr Dis Treat. (2018) 14:1545–59. 10.2147/NDT.S166730 29950839 PMC6011882

[18] Kruisdijk F Hopman-Rock M Beekman AT Hendriksen IJ. Personality traits as predictors of exercise treatment adherence in major depressive disorder: lessons from a randomised clinical trial. Int J Psychiatry Clin Pract. (2020) 24:380–6. 10.1080/13651501.2020.1787452 32657194

[19] Mokhtari S Shariat SV Ardebili ME Shalbafan M. Iranian students' attitudes toward premarital sex, marriage, and family in different college majors. J Am Colleg Health. (2022) 70:1186–94. 10.1080/07448481.2020.1789150 32672512

[20] Eissazade N Hemmati D Ahlzadeh N Shalbafan M Askari-Diarjani A Mohammadsadeghi H. Attitude towards migration of psychiatric trainees and early career psychiatrists in Iran. BMC Med Educ. (2021) 21:1–7. 10.1186/s12909-021-02926-y 34551745 PMC8459496

[21] Shariati B Shabani A Ariana-Kia E Ahmadzad-Asl M Alavi K Behbahani ZM. Drug attitude inventory in patients with bipolar disorder: Psychometric properties. Iran J Psychiatr Behav Sci. (2018) 12:e9831. 10.5812/ijpbs.983128257447

[22] Asadi M Rashedi V Khademoreza N Seddigh R Keshavarz-Akhlaghi A-A Ahmadkhaniha H. Medication adherence and drug attitude amongst male patients with the methamphetamine-induced psychotic disorder after discharge: a three months follow up study. J Psychoactive Drugs. (2022) 54:18–24. 10.1080/02791072.2021.1883778 33594958

[23] Sperber CM Samarasinghe SR Lomax GP. An upper and lower bound of the medication possession ratio. Pat Pref Adherence. (2017) 11:1469–78. 10.2147/PPA.S136890 28919719 PMC5590759

[24] Zamanzadeh V Ghahramanian A Rassouli M Abbaszadeh A Alavi-Majd H Nikanfar A-R. Design and implementation content validity study: development of an instrument for measuring patient-centered communication. J Caring Sci. (2015) 4:165–78. 10.15171/jcs.2015.017 26161370 PMC4484991

[25] Caqueo-Urízar A Urzúa A Miranda-Castillo C Irarrázaval M. Adherencia a la medicación antipsicótica en pacientes indígenas con esquizofrenia. Salud Mental. (2016) 39:303–10. 10.17711/SM.0185-3325.2016.035

[26] Winton-Brown TT Elanjithara T Power P Coentre R Blanco-Polaina P McGuire P. Five-fold increased risk of relapse following breaks in antipsychotic treatment of first episode psychosis. Schizophr Res. (2017) 179:50–6. 10.1016/j.schres.2016.09.029 27745754

[27] Rahimian Boogar I Moazedian A. Improvement of borderline personality disorder at different time frames of transference focused psychotherapy: a case report. Stu Med Sci. (2020) 31:255–66.

[28] Zargar Y Sajadi SF Mehrabizade Honarman M Arshadi N. Validation of the borderline personality features scale for children on students in Shiraz. Stu Med Sci. (2014) 25:338–52.

[29] Soltaninejad A Ashtiani AF Ahmadi K Yahaghi E Nikmorad A Karimi R. Structural equation model of borderline personality disorder, emotion-focused coping styles, impulsivity and suicide ideation in soldiers. J Police Med. (2012) 1:176–82. 10.30505/1.3.5

[30] Azadforouz S Shabani A Nohesara S Ahmadzad-Asl M. Non-compliance and related factors in patients with bipolar I disorder: a six month follow-up study. Iran J Psychiatr Behav Sci. (2016) 10:e2448. 10.17795/ijpbs-2448 27803718 PMC5087110

[31] Timäus C Meiser M Bandelow B Engel KR Paschke AM Wiltfang J. Pharmacotherapy of borderline personality disorder: what has changed over two decades? A retrospective evaluation of clinical practice. BMC Psychiatry. (2019) 19:1–11. 10.1186/s12888-019-2377-z 31830934 PMC6909459

